# Synthesis and Photophysical Properties of AIE-Type Carbazole-Capped Triphenylmethyl Organic Radicals Featuring Non-Aufbau Electronic Structure and Enhanced Photostability

**DOI:** 10.3390/molecules30061344

**Published:** 2025-03-17

**Authors:** Hazretomar Parida, Fudong Ma, Zunqi Liu, Zhaoze Ding, Obolda Ablikim

**Affiliations:** College of Chemistry and Chemical Engineering, Xinjiang Agricultural University, East Road No. 311, Urumqi 830052, China; phazretomar@126.com (H.P.); xjaumafud@163.com (F.M.); lzq@xjau.edu.cn (Z.L.); dzz13201290690@126.com (Z.D.)

**Keywords:** TTM radical, donor–acceptor (D-A•)-type molecule, AIE unit, non-Aufbau electronic structural

## Abstract

In this study, we report two novel donor–acceptor (D-A•)-type triphenylmethyl radicals, TTM-1TPE-2Cz and TTM-2TPE-2Cz, synthesized by integrating an aggregation-induced emission (AIE)-active 2-(1, 2, 2-triphenylethenyl)-9H-carbazole (TPE-2Cz) donor with tris(2,4,6-trichlorophenyl)methyl (TTM) radical core. Despite the AIE unit’s conventional ACQ-suppressing capability, both radicals exhibit complete emission quenching in solid/solution states but demonstrate 655 nm red emission in polymethyl methacrylate (PMMA)-doped films. Theoretical and experimental analyses reveal that the flexible TPE moiety unexpectedly enhances non-radiative decay while establishing a non-Aufbau electronic configuration through its strong electron-donating nature (−5.16 eV HOMO vs. −5.75 eV SOMO). Remarkably, these radicals achieve unprecedented photostability with half-lives (t₁/₂) 39,000- and 12,000-fold greater than pristine TTM, respectively. This work not only presents a synthetic strategy for stable radicals through non-Aufbau electronic engineering but also elucidates critical structure–property relationships between AIE units and radical photophysics.

## 1. Introduction

Since Gomberg’s seminal discovery of stable triphenylmethyl radicals in 1900 [1], triarylmethyl derivatives have remained the mainstay of room-temperature emissive radical research. While early studies predominantly focused on these systems due to their exceptional stability [2,3,4,5], recent advances have revealed emissive radical architectures beyond classical triarylmethyl frameworks, including boron-stabilized triphenylmethyl radical [6], dithiadiazolyl (DTDA) radical [7] and other organic radicals [8,9,10]. Currently, the most widely studied triphenylmethyl radicals include the following three types: the perchlorinated triphenylmethyl (PTM) luminescent radicals [2,11], the tri(2,4,6-trichlorophenyl)methyl (TTM) luminescent radicals [12,13,14], and the (3,5-dichloro-4-pyridyl-bis(2,4,6-trichlorophenyl)methyl (PyBTM) luminescent radicals [15,16,17,18] and other triarylmethyl-type radicals [19,20,21,22]. A landmark advancement occurred in 2006 when Juliá et al. [14,23] pioneered the synthesis of stable D-A•-type luminescent radical TTM-1Cz through C-N coupling of carbazole donor groups to the TTM core. Subsequent investigation established that such D-A• structures always exhibit enhanced higher photoluminescence quantum efficiency (PLQE) and better photostability [11,24,25]. Therefore, the synthesis of radicals with D-A• structure is crucial to improve the luminous efficiency of TTM-type radicals. The emission of those radicals has the intramolecular charge transfer (ICT) feature [26,27]. However, the fluorescence of D-A• radicals will be greatly weakened or even quenched in strong polar solvents.

**Scheme 1:**
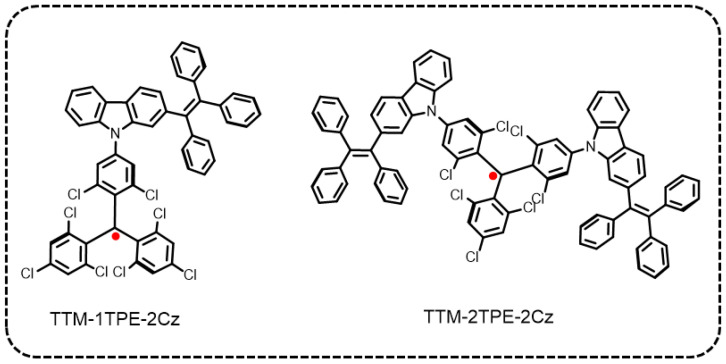
Molecular structure of TTM-1TPE-2Cz and TTM-2TPE-2Cz. The red dot represents unpaired electrons, a common notation for organic radicals in chemical structural representations.

The fluorescence quenching of traditional organic materials in the solid state or highly concentrated aggregated state can be attributed to the aggregation-caused quenching (ACQ) phenomenon caused by the close packing of the π-π bonds of organic luminescent materials in the solid state or highly concentrated aggregated state [28], which limits the practical application of organic luminescent materials. The unique property of aggregation-induced emission (AIE) molecules, which do not emit light or emit weakly in benign solvents but emit light in the aggregated state, endows them with significant advantages in fields such as biological probes [29], cell imaging [30], chemical sensing [31], and electroluminescence [32]. The majority of molecules with AIE properties contain arylalkenyl moieties. Tetraphenylethylene, as a typical structural unit for constructing AIE molecules, exhibits excellent photochemical and photophysical activity [33]. To address the fluorescence quenching of D-A• radicals in aggregated states while maintaining their high PLQE characteristics, we strategically selected the AIE-active unit TPE-2Cz as the donor. This design was motivated by two key considerations: (i) the non-alternating conformation of TPE-2Cz, which integrates a carbazole and TPE unit, could potentially disrupt π-π stacking to mitigate ACQ effects while preserving its high PLQE, and (ii) its multiple substitution sites allow systematic investigation of donor–acceptor spatial arrangements (Scheme 1). Specifically, we engineered two derivatives with varying TPE-2Cz numbers (TTM-1TPE-2Cz and TTM-2TPE-2Cz) to investigate: (i) how steric hindrance from the AIE moiety affects radical stability and its PLQE; (ii) whether increased donor substitution modulates the (singly occupied molecular orbital, SOMO; highest occupied molecular orbital, HOMO) SOMO-HOMO energy arrangement for enhanced ICT emission.

Contrary to expectations from carbazole-TTM systems, the flexible TPE-2Cz linkage unexpectedly accelerated non-radiative decay, suggesting critical rigidity requirements for D-A• radical design that differ fundamentally from closed-shell AIE systems. This paradox indicates the need for new design rules when integrating AIE motifs with open-shell systems.

## 2. Results and Discussion

### 2.1. Fluorescence and UV–Vis Absorption Spectra

**Figure 1:**
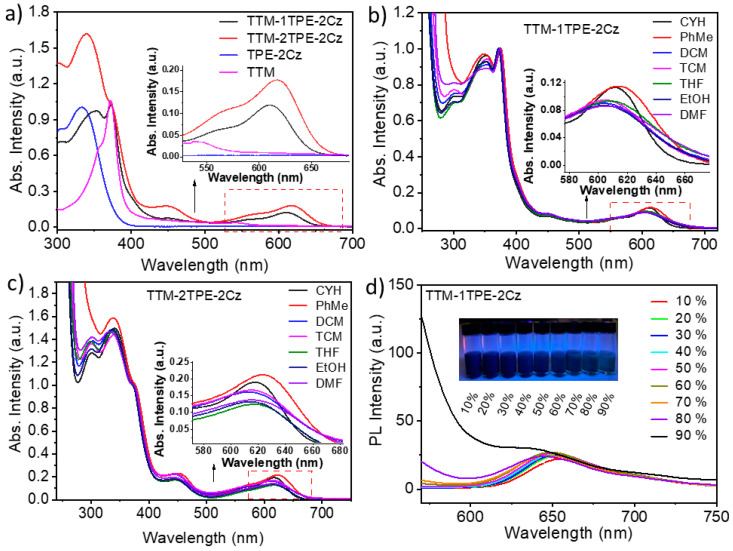
(**a**) UV–vis absorption spectra of radicals in cyclohexane. (**b**,**c**) UV–vis absorption spectra of the radical TTM-1TPE-2Cz and TTM-2TPE-2Cz in different polar solvents. (**d**) Photoluminescence (PL) spectra (λ_ex_ = 450 nm) of radical TTM-1TPE-2Cz with different ratios in cyclohexane/polydimethylsiloxane mixed solvent system (1 × 10^−3^ mol/L), the insect shows the related photographs.

First, we measured the absorption and emission spectra of the TTM-1TPE-2Cz and TTM-2TPE-2Cz radicals. As shown in Figure 1a, both radicals exhibit characteristic absorption bands in a cyclohexane solution. In the short-wavelength region (300–400 nm), two distinct peaks are observed at 350 nm and 375 nm. The former absorption peak with fine structure is attributed to the TPE-2Cz unit, while the 375 nm absorption peaks can be ascribed to the π-π* characteristic local transition from the TTM moiety. In contrast, the broad absorption spanning 550–700 nm is assigned to intramolecular charge transfer (ICT) state absorption from the donor (TPE-2Cz) to the TTM unit, which was further proved by DFT calculations (see Section 2.2). This ICT behavior is common for many D-A•-type organic radicals [5,11]. To evaluate solvent effects, we systematically measured the absorption spectra of the radical in different polar solvents (Figure 1b,c). Notably, both radicals maintained nearly identical absorption profiles in different polar solvents, indicating minimal solvent-polarity dependence of their ground-state electronic configurations.

To our surprise, neither TTM-1TPE-2Cz nor TTM-2TPE-2Cz exhibited fluorescence in solution or the solid state after coupling the TPE-2Cz monomer to the TTM radical. We attribute this phenomenon primarily to enhanced non-radiative vibration relaxation induced by the flexible TPE-2Cz unit. Given the well-documented susceptibility of D-A• radicals to polarity-driven fluorescence quenching, our preliminary attempts to acquire measurable emission signals from THF/water mixtures resulted in complete emission quenching, necessitating alternative solvent engineering strategies. Thus, to explore the AIE performance of the target radicals, we prepared cyclohexane/polydimethylsiloxane mixtures (1 × 10^−3^ mol/L) with varying solvent ratios and measured their PL spectra. The viscous polydimethylsiloxane and non-polar cyclohexane were strategically combined as co-solvents to suppress molecular motion, promote aggregation, and avoid polarity-induced fluorescence quenching—a critical design consideration for D-A•-type radicals prone to solvent-driven emission loss. As shown in Figure 1d, even at a high polydimethylsiloxane content *f*_w_ = 90%, the radicals still did not show significant fluorescence emission peaks, indicating that introducing the well-known AIE-active unit TPE-2Cz moiety cannot suppress the ACQ in those TTM-based radicals.

**Figure 2:**
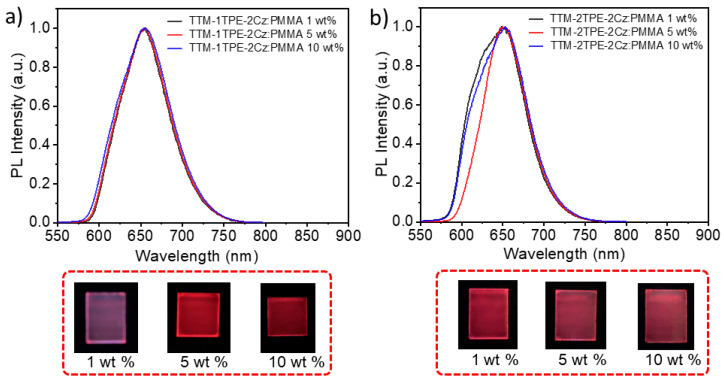
PL spectra (λ_ex_ = 375 nm) and corresponding photographs (under 365 nm UV irradiation) of (**a**) TTM-1TPE-2Cz and (**b**) TTM-2TPE-2Cz-doped in PMMA thin films; both sets of films were prepared with dopant concentrations of 1 wt%, 5 wt%, and 10 wt%.

To further investigate emission behavior, we selected polymethyl methacrylate (PMMA) as the host material and doped the radical molecules with different ratios (1 wt%, 5 wt%, and 10 wt%) and studied their photophysical properties. Intriguingly, both radical-doped films displayed red fluorescence (λ_em_ ≈ 655 nm) under UV light excitation, as depicted in Figure 2a,b.

**Figure 3:**
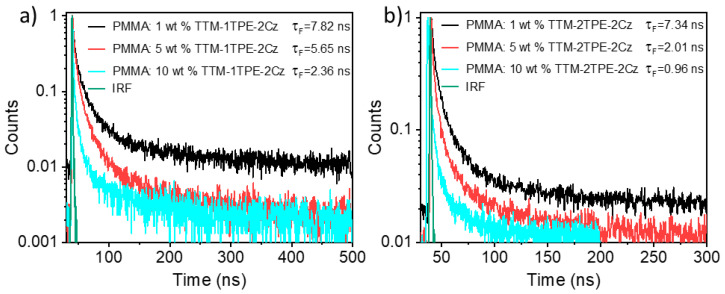
Fluorescence lifetime of (**a**) TTM-1TPE-2Cz and (**b**) TTM-2TPE-2Cz films with different concentrations (1 wt%, 5 wt%, 10 wt%) in PMMA (λ_ex_ = 375 nm).

Furthermore, we also measured the fluorescence quantum yield (Φ_F_) and fluorescence lifetime (τ) of PMMA-doped films containing TTM-1TPE-2Cz and TTM-2TPE-2Cz at varying concentrations (1–10 wt%). As shown in Figure 3a,b and Table 1, both radicals exhibited concentration-dependent Φ_F_ reduction: for TTM-1TPE-2Cz, Φ_F_ decreased from 4.9% (1 wt%) to 5.0% (5 wt%) and 1.3% (10 wt%), while TTM-2TPE-2Cz showed a parallel decline from 3.6% to 2.8% and 0.7%. Notably, the fluorescence lifetimes displayed distinct non-monotonic trends: for TTM-1TPE-2Cz, τ values were 7.82 ns (1 wt%), 5.65 ns (5 wt%), and 2.36 ns (10 wt%), whereas TTM-2TPE-2Cz exhibited τ values of 7.34 ns, 2.01 ns, and 0.96 ns at corresponding concentrations. Rate constant analysis revealed dominant non-radiative pathways: the non-radiative transition rates (*k_nr_* = 1.22–4.20 × 10^8^ s^−1^ for TTM-1TPE-2Cz; 1.32–10.3 × 10^8^ s^−1^ for TTM-2TPE-2Cz) exceeded radiative rates (*k_r_* = 0.626–0.551 × 10^7^ s^−1^ and 0.490–0.729 × 10^7^ s^−1^, respectively) by 10–200-fold, spanning two orders of magnitude. The resulting *k_nr_/k_r_* ratios (19–76 for TTM-1TPE-2Cz; 27–141 for TTM-2TPE-2Cz) conclusively demonstrate that non-radiative decay dominates the excited-state dynamics, rationalizing the overall weak luminescence efficiency across all doped systems.

The above results indicated that, contrary to conventional D-A• systems where structural rigidity ensures efficient emission [11,24,27], the flexible TPE-2Cz donor in our derivatives introduces vibrational dissipation channels that accelerate non-radiative relaxation. This is quantified by the *k_nr_*/*k_r_* ratios exceeding 10^1^–10^2^ (Table 1), consistent with the energy gap law for non-radiative decay in vibrationally active systems [34]. The observed concentration-dependent lifetime reduction (Figure 3a,b) further reflects escalating quenching effects. While close packing at 10 wt% partially restricts intramolecular motion, intermolecular fluorescent quenching becomes significant (Φ_F_), decreasing both τ and Φ_F_.

### 2.2. Density Functional Theory (DFT) Calculations

To gain an in-depth understanding of the frontier orbital electronic structure and photophysical properties of TTM-1TPE-2Cz and TTM-2TPE-2Cz radicals, we performed density functional theory (DFT) calculations using UB3LYP/6-31G (d,p) method. As shown in Figure S1, the dihedral angles between the TTM core and TPE-2Cz donor moiety are 49.1° (TTM-1TPE-2Cz) and 50.4° (TTM-1TPE-2Cz), respectively, indicating effective conjugation between TTM and the electron-donating group TPE-2Cz.

**Figure 4:**
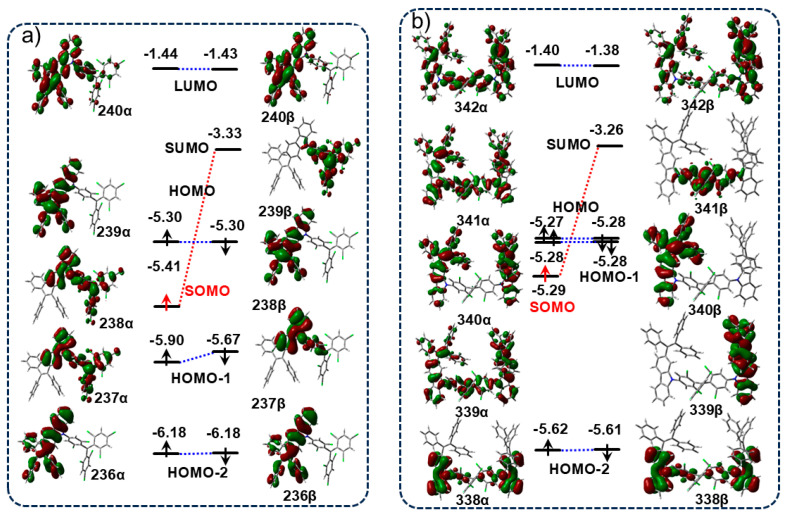
Frontier orbital energy levels and electron cloud distribution of (**a**) TTM-1TPE-2Cz and (**b**) TTM-2TPE-2Cz.

The singly occupied molecular orbital (SOMO) and singly unoccupied molecular orbital (SUMO) of the TTM are mainly localized on the radical TTM unit (Supplementary Materials Figure S2). In contrast, the highest doubly occupied molecular orbital (HOMO) of both radicals TTM-1TPE-2Cz and TTM-2TPE-2Cz is mainly distributed on the electron-donating substituent TPE-2Cz (Figure 4a,b). Notably, the strong electron-donating capability of TPE-2Cz elevates its HOMO energy level to −5.16 eV (Figure S2), which is 0.59 eV higher than the SOMO energy level of the TTM unit (−5.75 eV). This results in the HOMO energy level of the entire radical molecule being higher than that of the SOMO, presenting the SOMO-HOMO-inverted non-Aufbau electronic structure of the radicals [11,35,36]. Considering that the TPE-2Cz unit has stronger electron-donating abilities, it is reasonable that the energy level of HOMO is higher than that of SOMO.

Furthermore, the time-dependent density functional theory (TD-DFT) calculations (Supporting Information Section S2) were performed to gain deeper insights into the excited-state characteristics. Calculation results revealed that the doublet excited state (D_1_) of both TTM-1TPE-2Cz and TTM-2TPE-2Cz radicals originated from 238β-239β and 340β-341β electronic transitions, with corresponding oscillator strengths (*f*) of 0.0013 and 0.0008, respectively. Notably, these values are significantly lower than that of the weakly emissive parent TTM radical (*f =* 0.0223). Although lower energy band absorption profiles of TTM-1TPE-2Cz and TTM-2TPE-2Cz resemble that of TTM-1Cz, the introduction of a flexible TPE framework appears to significantly enhance the non-radiative decay pathway. This comparative analysis suggests that the incorporation of AIE-active units into the radical core structure ultimately suppresses luminescent properties through the activation of competitive non-radiative relaxation processes.

### 2.3. Cyclic Voltammetry (CV) Measurements

**Figure 5:**
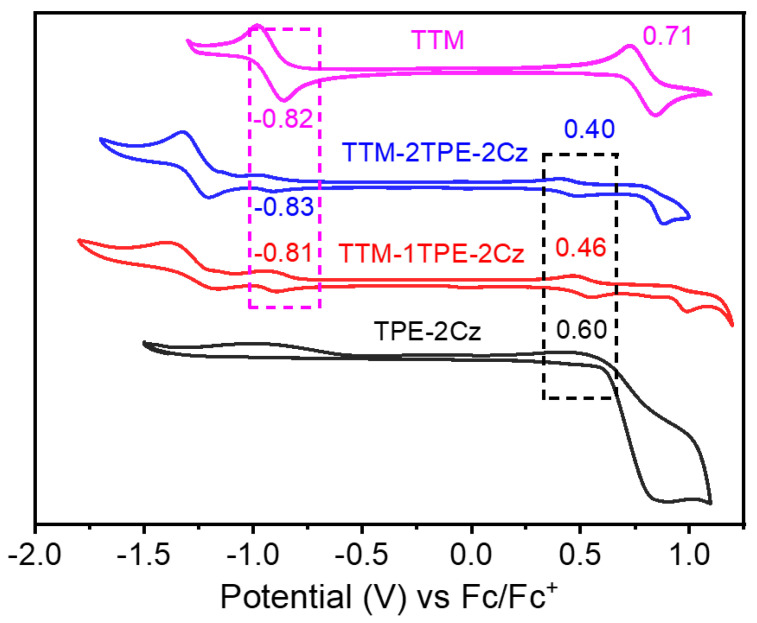
Cyclic voltammetry (CV) curves of TTM-1TPE-2Cz, TTM-2TPE-2Cz, TTM, and TPE-2Cz.

To further verify the non-Aufbau electronic structure, we conducted cyclic voltammetry (CV) tests on both radicals. As depicted in Figure 5, the first reduction potentials of TTM-1TPE-2Cz and TTM-2TPE-2Cz were −0.81 V and −0.83 V, respectively, which are very close to the reduction potential of the parent TTM radical (−0.82 V). This alignment confirms that the (SUMO) of the radicals mainly originated from the TTM unit. According to the calculation of the optical energy level difference, the SUMO energy levels of the two radicals, calculated from the optical bandgap (*E_g_*), are −3.34 eV and −3.32 eV, respectively, which agree well with theoretical predictions (−3.33 eV and −3.26 eV). In contrast, the first oxidation potentials of TTM-1TPE-2Cz (0.46 V) and TTM-2TPE-2Cz (0.40 V) are significantly lower than that of TTM (0.71 V) but align closely with the oxidation potential of the TPE-2Cz donor (0.60 V). We assumed that those first oxidation peaks mainly originate from the donor unit TPE-2Cz. The results combined with theoretical calculations indicate that the energy level of the HOMO is higher than that of the SOMO [11,36]. According to CV oxidation potentials of the radicals TTM-1TPE-2Cz and TTM-2TPE-2Cz, the calculated energy level of HOMO is −5.26 eV and −5.20, which is also supported by DFT calculation (−5.30 eV and −5.28 eV) (see Table 2).

### 2.4. Stability of Radicals

**Figure 6:**
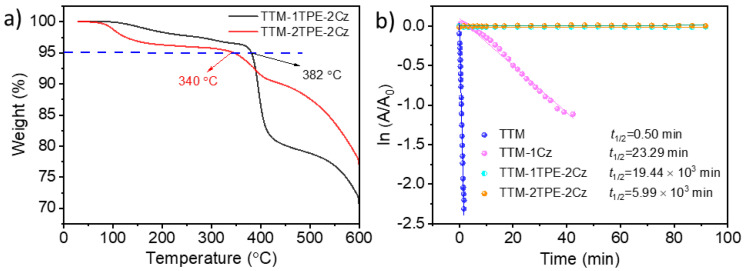
(**a**) TGA curves and (**b**) photostability of radicals.

To study the thermal stability of the radicals, thermogravimetric analysis (TGA) was performed on TTM-1TPE-2Cz and TTM-2TPE-2Cz. As shown in Figure 6a, the temperatures corresponding to 5% mass loss for TTM-1TPE-2Cz and TTM-2TPE-2Cz were 382 °C and 340 °C, respectively. These results indicate that TTM-1TPE-2Cz exhibits significantly higher thermal stability than TTM-2TPE-2Cz. In addition to thermal stability, photostability is one of the important parameters of organic radicals. To assess this property, the decay of the absorption peak centered at 375 nm was monitored for both radicals in cyclohexane solution under 365 nm UV lamp irradiation, with identical concentrations and instrument settings. For comparison, the well-characterized radical TTM and TTM-1Cz were also tested under the same conditions. As shown in Figure 6b, the calculated halftime (t_1/2_) of the TTM-1TPE-2Cz and TTM-2TPE-2Cz in cyclohexane solutions were 19.44 × 10^3^ min and 5.99 × 10^3^ min, respectively. These values were nearly 39,000 times and 12,000 times higher than that of TTM (0.5 min) and 850 times and 260 times higher than that of TTM-1Cz (23.29 min), respectively.

## 3. Materials and Methods

### 3.1. Preparation of TPE-2Cz, TTM, TTM-1TPE-2Cz, and TTM-2TPE-2Cz

**Scheme 2:**
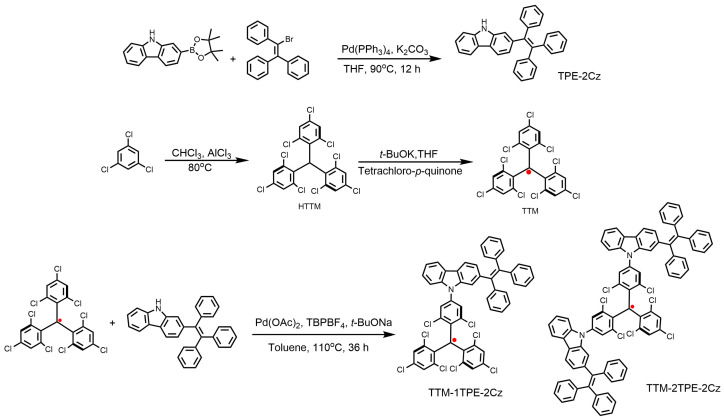
Synthetic procedure of TPE-2Cz and radical TTM, TTM-1TPE-2Cz, and TTM-2TPE-2Cz. The red dot represents unpaired electrons, a common notation for organic radicals in chemical structural representations.

Radical TTM [37] and TPE-2Cz [38] were synthesized according to the previous work (Scheme 2).

TPE-2Cz: In a 250 mL polymerization flask, 0.99 g (2.95 mmol) of triphenylethylene bromide (TPE-Br), 0.62 g (2.11 mmol) of 9*H*-carbazole-2-yl pinacol boronate were dissolved in THF (30 mL), and then 2 M potassium carbonate (10 mL) solution was added. Finally, 0.29 g (0.69 mmol) of Pd (PPh_3_)_4_ was added, and the mixture was stirred and heated at 90 °C for 12 h. The crude reaction product was cooled to room temperature and extracted with CH_2_Cl_2_. The organic phase was collected, and the solvent was evaporated. The crude product was separated by column chromatography using petroleum ether–dichloromethane = 4:1 (volume ratio). Finally, 0.34 g of white powder (TPE-2Cz) was obtained with a yield of 38%. For the IR, NMR (400 MHz, CDCl_3_), and MS (MALDI-TOF), please see the Supporting Information (Figures S3–S5).

TTM-1TPE-2Cz and TTM-2TPE-2Cz: Under the nitrogen atmosphere, 0.88 g (1.6 mmol) of tris(2,4,6-trichlorophenyl)methyl radical (TTM), 0.67 g (1.6 mmol) of TPE-2Cz, 0.084 g (0.29 mmol) of tris(tert-butyl)phosphine tetrafluoroborate (TBPBF_4_), 0.021 g (0.094 mmol) of palladium acetate (Pd(OAc)_2_), and 0.15 g (1.56 mmol) of sodium tert-butoxide (*t*-BuONa) were dissolved in toluene solution (40 mL), then and the mixture was heated and stirred at 110 °C for 36 h. The crude reaction product was cooled to room temperature, extracted with CH_2_Cl_2_, and concentrated by rotary evaporation. The crude product was separated by column chromatography using petroleum ether–dichloromethane = 8:1 (volume ratio). Finally, 0.27 g of dark green solid TTM-1TPE-2Cz and 0.36 g TTM-2TPE-2Cz were obtained, with yields of 18% and 17%, respectively. HRMS (*m/z*) and EPR, please see the Supporting Information (Figures S6 and S7): TTM-1TPE-2Cz: calculated for [M]^+^ 937.7; C_51_H_28_C_l8_N found [M]^+^ 937.9; HRMS calculated for [M]^+^ 933.9724; C_51_H_28_C_l8_N found [M]^+^ 933.9746; TTM-2TPE-2Cz: calculated for [M]^+^ 1321.6; C_83_H_50_C_7_N_2_ found [M]^+^ 1321.1; HRMS calculated for [M+H]^+^ 1320.1967; C_83_H_50_C_7_N_2_ found [M+H]^+^ 1320.1788.

### 3.2. Materials

Triphenylethylene bromide (TPE-Br), 9*H*-carbazole-2-boronic acid pinacol ester, tetrakis(triphenylphosphine)palladium, 1,3,5-trichlorobenzene, anhydrous aluminum trichloride (AlCl_3_), tris(tert-butyl)phosphine tetrafluoroborate (TBPBF_4_), palladium acetate (Pd(OAc)_2_), sodium tert-butoxide (*t*-BuONa), potassium tert-butoxide (*t*-BuOK), and tetrachloro-p-benzoquinone were purchased from Adamas Reagent Co., Ltd. (Shanghai, China) Tetrahydrofuran (THF), potassium carbonate, dichloromethane (CH_2_Cl_2_), petroleum ether, toluene, chloroform (CHCl_3_), hydrochloric acid (HCl), and anhydrous sodium sulfate were all purchased from Tianjin Xinbaite Chemical Co., Ltd. (Tianjin, China). The reagents used are analytical grade. All measurements were taken at room temperature.

### 3.3. Methods

The ^1^H-NMR spectra were recorded with a VARIAN 500 spectrometer (Agilent Technologies, Santa Clara, CA, USA) operating at 400 MHz, using deuterated chloroform (CD_3_Cl) as solvent at 298 K. The mass spectra of the radical were recorded on LCMS-IT-TOF (Shimadzu Corporation, Kyoto, Japan) for high resolution. Fourier transform infrared (FT-IR) spectra were recorded using a Bruker VERTEX 70 (Bruker Corporation, Billerica, MA, USA). Ultraviolet–visible (UV–vis) absorption spectra of the radicals were recorded on a Shimadzu UV-2600 spectrophotometer (Shimadzu Corporation, Kyoto, Japan). Fluorescence spectra of the radicals were performed using an Agilent G9800A spectrophotometer (Agilent Technologies, Santa Clara, CA, USA). EPR spectra were recorded on a Bruker ELEXSYSII E500 CW-EPR spectrometer (Bruker Corporation, Billerica, MA, USA) at ambient atmosphere in dichloromethane. The CV curves of the radical measurements were performed using a CHI760 electrochemical workstation (CH Instruments, Inc., Shanghai, China). The photostability of the radicals was achieved by measuring the decay of 375 nm centered UV–vis spectra under 365 nm UV lamp irradiation (Hangzhou Qiwei Corporation, Hangzhou, China). The TGA test results were obtained using Q50 thermogravimetric analyzer (TA Instruments, New Castle, DE, USA). The DFT and TD-DFT calculations were performed using the Gaussian 09 A.1 commercial software [39].

## 4. Conclusions

In conclusion, we have synthesized two novel D-A•-type organic radicals by integrating the carbazole-based AIE unit with the TTM radical core. Systematic photophysical studies and DFT calculations reveal that closed-shell AIE-active units, effective in suppressing aggregation-caused quenching (ACQ) in conventional systems, fail to mitigate ACQ in these TTM-based radicals. Contrary to expectations, the introduced AIE unit not only fails to suppress ACQ in TTM-1TPE-2Cz and TTM-2TPE-2Cz but also accelerates non-radiative decay rates, likely due to enhanced vibrational freedom from the flexible TPE moiety. Nevertheless, the strong electron-donating ability of the TPE-2Cz endows these radicals with non-Aufbau electronic structure and more photostability. Notably, the calculated t_1/2_ of the radicals TTM-1TPE-2Cz and TTM-2TPE-2Cz in cyclohexane solution were 850-fold and 260-fold higher than that of the well-characterized TTM-1Cz (23.29 min), respectively. This work not only expands the family of stable organic radicals but also provides critical insights into manipulating the photophysical properties of functional materials.

## Figures and Tables

**Table 1:** Photophysical parameters of TTM-1TPE-2Cz and TTM-2TPE-2Cz doped in PMMA films.

Radical	Doping Ratio (wt%)	λ_e_^a^ (nm)	τ ^b^ (ns)	Ф_F_^c^ (%)	*k_r_*^d^ (×10^7^ s^−1^)	*k_nr_*^e^ (×10^8^ s^−1^)	*k_nr_* */k_r_*
	1	656	7.82	4.9	0.626	1.22	19.5
TTM-1TPE-2Cz	5	655	5.65	5.0	0.885	1.68	19.0
	10	655	2.36	1.3	0.551	4.20	76.2
	1	655	7.34	3.6	0.490	1.32	26.7
TTM-2TPE-2Cz	5	656	2.01	2.8	1.39	4.84	34.8
	10	656	0.96	0.7	0.729	10.3	141.1

Note: ^a^ the maximum emission wavelength; ^b^ fluorescence lifetime; ^c^ fluorescence quantum yield; ^d^ radiative transition rate; ^e^ non-radiative transition rate.

**Table 2:** Summarized frontier orbital energy levels of TTM-1TPE-2Cz and TTM-2TPE-2Cz.

Radical	λ_Onset_/ nm	HOMO Cal ^a^/CV ^b^ (eV)	SOMO Cal ^a^/CV ^b^ (eV)	SUMO Cal ^a^/*E*_g_^c^ (eV)	LUMO Cal ^a^/CV ^b^ (eV)	*_Δ_E* Cal ^a^/*E*_g_ ^d^ (eV)
TTM-1TPE-2Cz	645	−5.30/−5.26	−5.40/-	−3.33/−3.34	−1.44/-	2.07/1.92
TTM-2TPE-2Cz	658	−5.28/−5.20	−5.29/-	−3.26/−3.32	−1.40/-	2.03/1.88

Note: ^a^ achieved from theoretical calculation; ^b^ achieved from cyclic voltammetry curves; ^c^ SUMO energy was calculated from the optical energy gap and HOMO (*E*_SUMO_ = *E*_HOMO_ + *E*_g_); ^d^ optical energy gap.

## Data Availability

Data will be made available on request.

## Supplementary Materials

The following supporting information can be downloaded at https://www.mdpi.com/article/10.3390/molecules30061344/s1, Figure S1. Molecular configuration of (a) TTM-1TPE-2Cz and (b) TTM-2TPE-2Cz calculated by UB3LYP/6-31G (d,p) methods; Figure S2. Frontier orbital energy levels and electron cloud distribution of (a) TTM and (b) TPE-2Cz. Figure S3. NMR spectra of the monomer TPE-2Cz, top for ^1^H-NMR, below for ^13^C-NMR; Figure S4. Structural characterization of monomer TPE-2Cz; Figure S5. Infrared spectra of TPE-2Cz, TTM-1TPE-2Cz, and TTM-2TPE-2Cz; Figure S6. Mass spectra of the radicals TTM-1TPE-2Cz and TTM-2TPE-2Cz; Figure S7. Electron paramagnetic resonance (EPR) spectra of TTM-1TPE-2Cz (a) and TTM-2TPE-2Cz (b) in CH_2_Cl_2_ at room temperature.
