# Supplementary material for: Synthesis and Photophysical Properties of AIE-Type Carbazole-Capped Triphenylmethyl Organic Radicals Featuring Non-Aufbau Electronic Structure and Enhanced Photostability
Source: Molecules. 2025 Mar 17;30(6):1344. doi: 10.3390/molecules30061344 (PMC11944396; doi:10.3390/molecules30061344)
Supplement: Supplementary file 1 [file molecules-30-01344-s001.zip › molecules-3511954-supplementary.pdf]

Supporting Information

Synthesis and Photophysical Properties of AIE-Type Carbazole-Capped Triphenylmethyl Organic Radicals Featuring Non-Aufbau Electronic Structure and Enhanced Photostability

Parida Hazretomar, Fudong Ma, Zunqi Liu, Zhaoze Ding, Ablikim Obolda*

Table of contents

Section 1. Characterization of the compounds 2

Section 2.TD-DFT calculation..7

Section 1. Characterization of the compounds

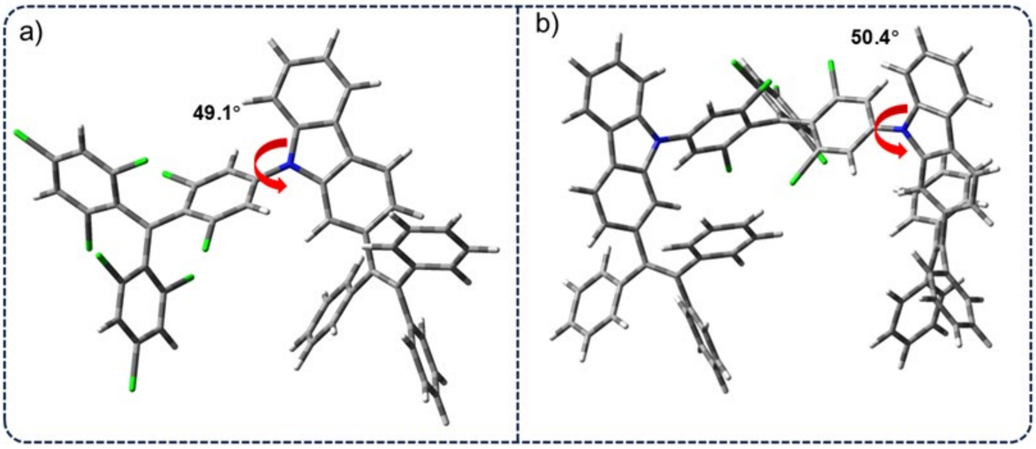

Figure S1. Molecular configuration of (a) TTM-1TPE-2Cz and (b) TTM-2TPE-2Cz calculated by UB3LYP/6-31G (d, p) methods.

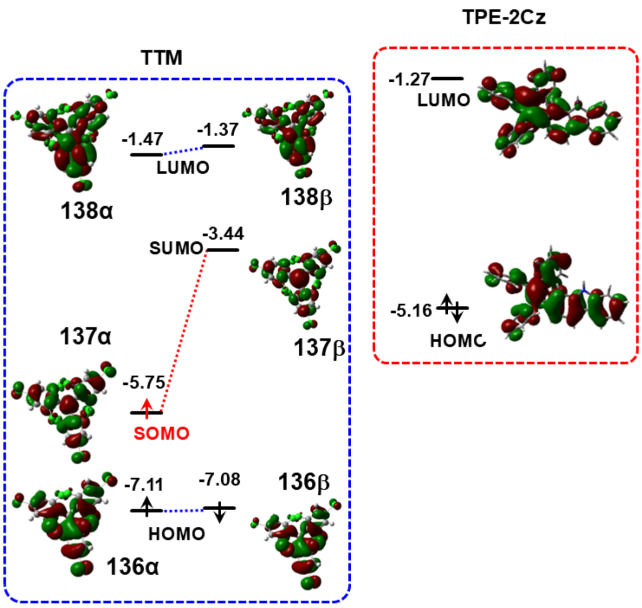

Figure S2. Frontier orbital energy levels and electron cloud distribution of (a) TTM and (b) TPE-2Cz.

TPE-2Cz: ^{1}H NMR (400 MHz, $CDCl_3$) δ 7.95 (d, J = 7.8 Hz, 1H), 7.80 – 7.73 (m, 2H), 7.38 – 7.28 (m, 2H), 7.17 (td, J = 6.2, 3.1 Hz, 1H), 7.17 – 6.99 (m, 16H), 6.92 (dd, J = 8.1, 1.5 Hz, 1H). ^{13}C NMR ($CDCl_3$, 101 MHz): δ 144.24, 144.00, 141.80, 141.58, 140.85, 139.87, 139.27, 131.49, 131.46, 131.39, 127.70, 127.68, 127.64, 126.41, 126.39, 126.34, 125.67, 123.48, 123.21, 121.81, 120.24, 119.44, 119.39, 113.60, 110.48.

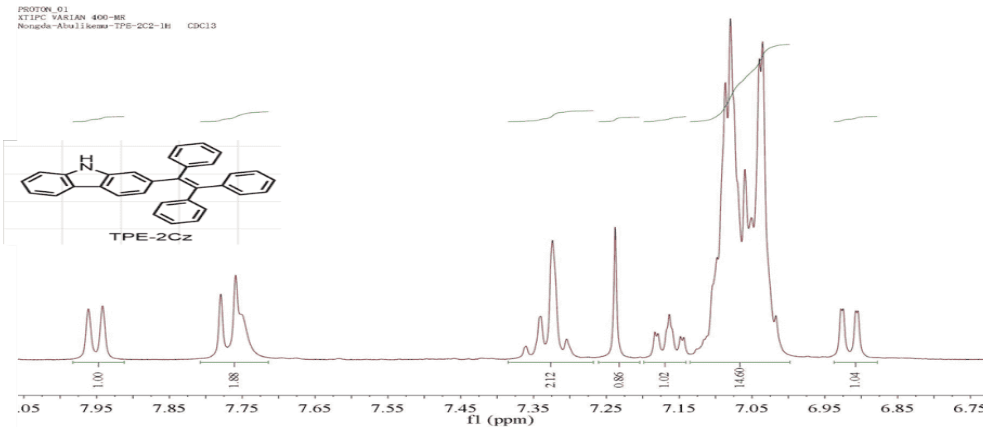

^{1}H-NMR spectrum (400 MHz) of TPE-2Cz in $CDCl_3$ at 298K

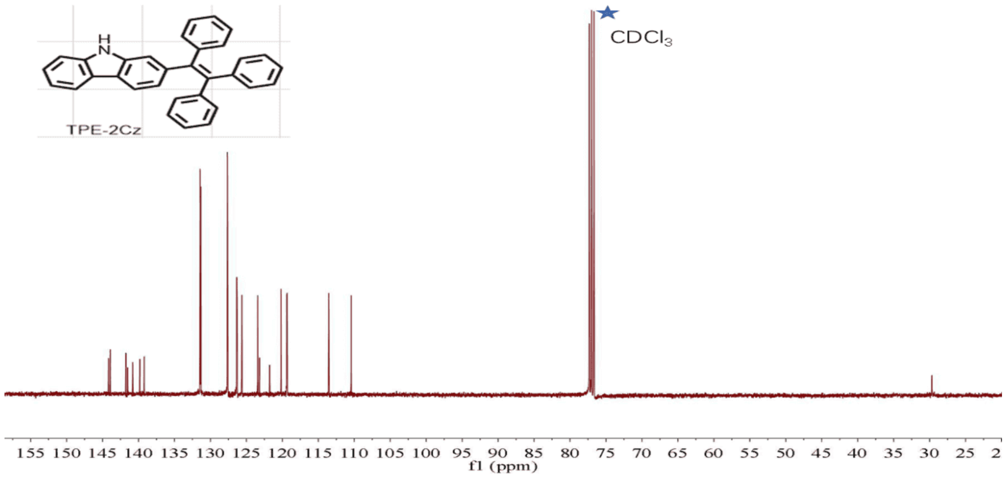

Figure S3. NMR spectra of the monomer TPE-2Cz, top for ^{1}H-NMR, below for ^{13}C-NMR

TPE-2Cz-MS (MALDI-TOF) Calculated for $C_{32}H_{23}N$ $[M]^+$: 421.54, found: $[M]^+$: 421.395.

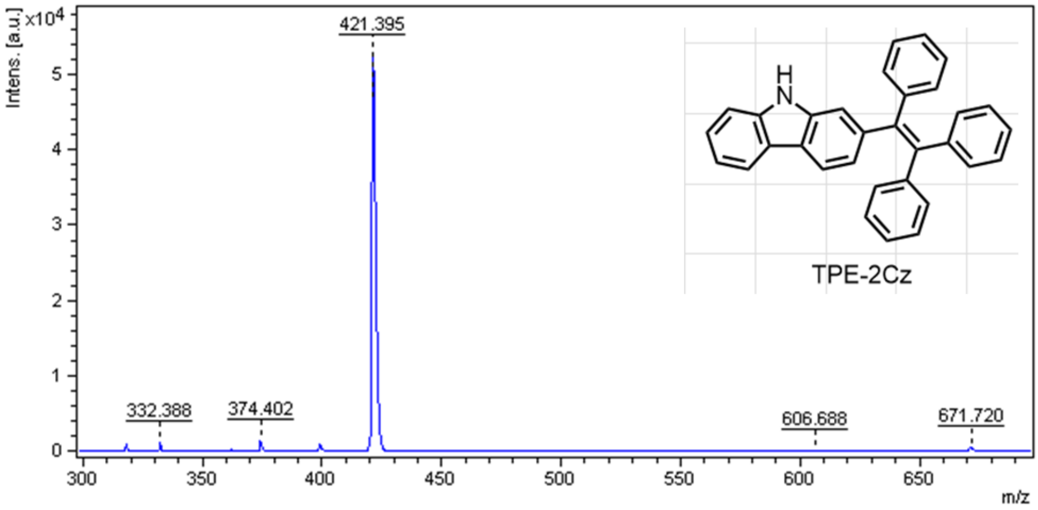

Figure S4. Structural characterization of monomer TPE-2Cz.

IR (cm^{-1}, s, strong; m, medium; w, weak): 3409 s, ν(–N-H); 3050 w, ν(–C-H); 1590 s, (C=C).

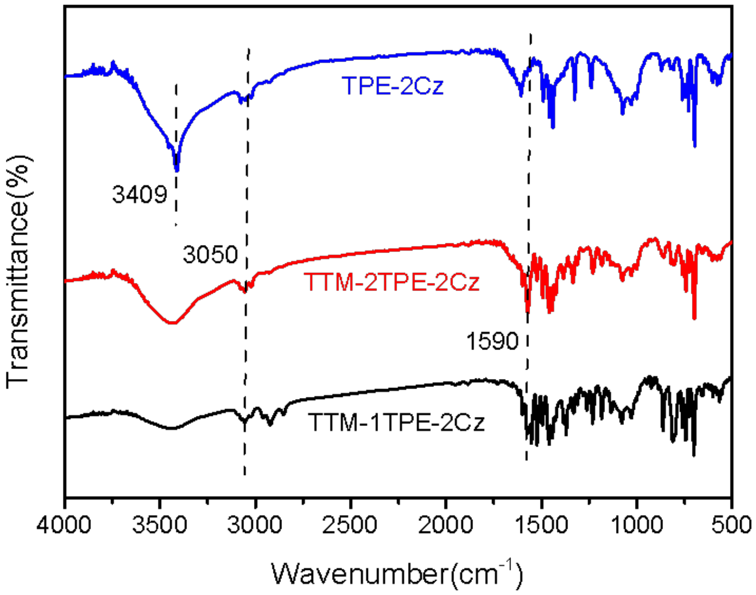

Figure S5. Infrared spectra of TPE-2Cz, TTM-1TPE-2Cz and TTM-2TPE-2Cz.

TTM-1TPE-2Cz-HRMS (m/z) Calculated for $C_{51}H_{28}Cl_8N$ $[M]^+$: 933.9724; found: $[M]^+$ 933.9746.

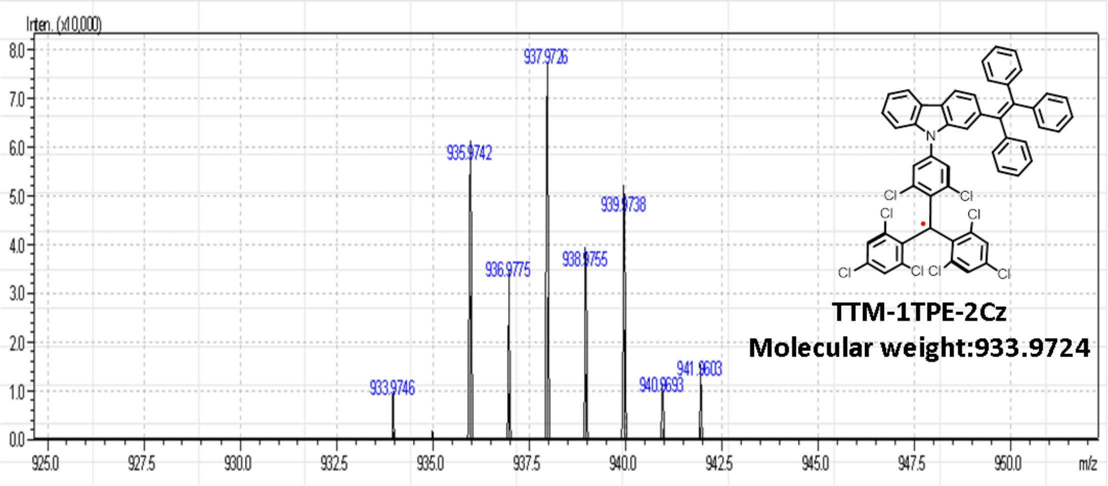

TTM-2TPE-2Cz-HRMS (m/z) Calculated for $C_{83}H_{50}C_7N_2$ requires $[M+H]^+$: 1320.1788; found: $[M+H]^+$: 1320.1967

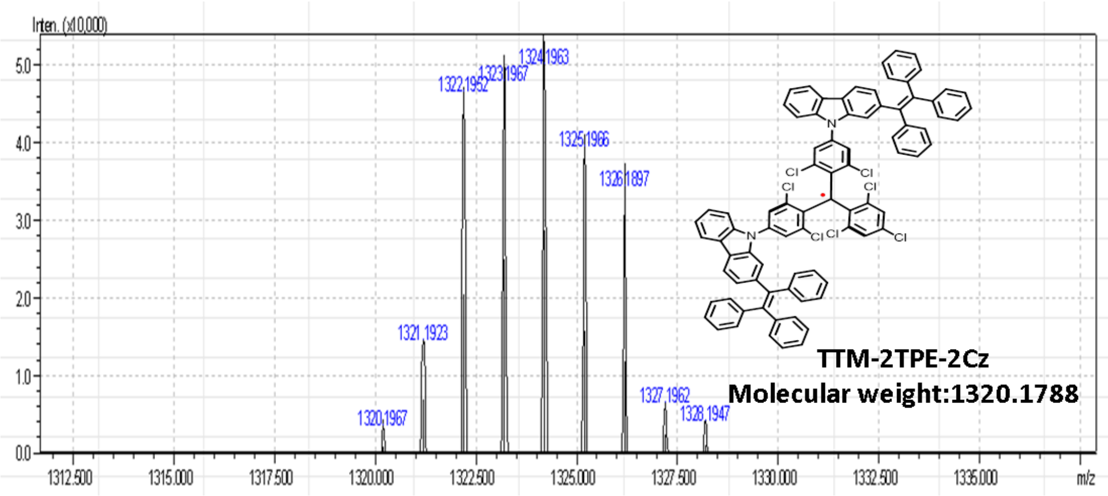

Figure S6. Mass spectra of the radicals TTM-1TPE-2Cz and TTM-2TPE-2Cz.

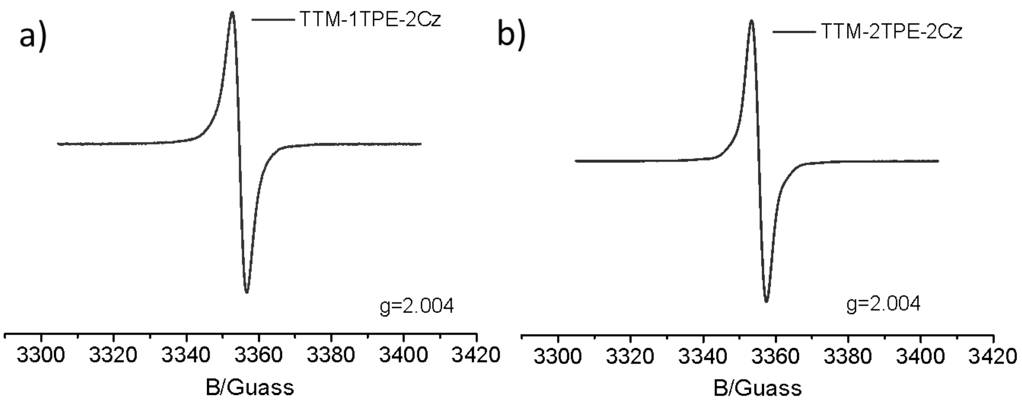

Figure S7. Electron paramagnetic resonance (EPR) spectra of TTM-1TPE-2Cz (a) and TTM-2TPE-2Cz (b) in CH_2Cl_2 at room temperature.

Section 2. TD-DFT calculation

TTM (Optimized ground state structure, UB3LYP/6-31G (d, p))

Excited State 1: 2.215-A 2.6552 eV 466.95 nm f=0.0223 S**2>=0.976

137A ->139A 0.30021

133B ->137B 0.22025

135B ->137B 0.11684

136B ->137B 0.87492

This state for optimization and/or second-order correction.

Total Energy, E(TD-HF/TD-KS) = -4869.25504405

Copying the excited state density for this state as the 1-particle RhoCI density.

Excited State 2: 2.215-A 2.6556 eV 466.87 nm f=0.0223 S**2>=0.976

137A ->138A 0.30019

132B ->137B -0.22002

135B ->137B 0.87488

136B ->137B -0.11716

Excited State 3: 2.101-A 2.9287 eV 423.35 nm f=0.0064 <S**2>=0.853

137A ->140A -0.15345

133B ->137B 0.23849

134B ->137B 0.93905

Excited State 4: 2.124-A 2.9297 eV 423.19 nm f=0.0030 <S**2>=0.878

137A ->139A 0.18059

133B ->137B 0.86537

134B ->137B -0.25744

135B ->137B -0.11317

136B ->137B -0.29529

Excited State 5: 2.127-A 2.9298 eV 423.18 nm f=0.0028 <S**2>=0.881

137A ->138A -0.18602

132B ->137B 0.89629

135B ->137B 0.30403

136B ->137B -0.12136

Excited State 6: 2.357-A 3.0872 eV 401.61 nm f=0.0000 <S**2>=1.138

135A ->138A -0.14918

136A ->139A 0.14922

137A ->146A -0.24504

131B ->137B 0.87169

134B ->140B -0.10798

Excited State 7: 2.371-A 3.3457 eV 370.58 nm f=0.1795 <S**2>=1.156

133A ->140A -0.11951

134A ->142A 0.10655

137A ->138A 0.72386

137A ->139A -0.33419

131B ->138B 0.10646

132B ->137B 0.32377

135B ->137B -0.27209

136B ->137B 0.11898

Excited State 8: 2.371-A 3.3459 eV 370.56 nm f=0.1799 <S**2>=1.155

132A ->140A -0.11943

134A ->141A 0.10679

137A ->138A 0.33423

137A ->139A 0.72391

131B ->139B 0.10657

133B ->137B -0.32376

135B ->137B -0.11895

136B ->137B -0.27216

Excited State 9: 2.206-A 3.5833 eV 346.01 nm f=0.0110 <S**2>=0.966

137A ->140A 0.95748

134B ->137B 0.17197

Excited State 10: 3.250-A 3.6827 eV 336.67 nm f=0.0000 <S**2>=2.390

132A ->138A	-0.15090
132A ->141A	0.19845
132A ->142A	-0.10256
133A ->139A	0.15104
133A ->141A	0.10285
133A ->142A	0.19833
134A ->140A	-0.32060
135A ->138A	0.17287
135A ->141A	0.10148
135A ->142A	-0.15256
136A ->139A	-0.17283
136A ->141A	0.15243
136A ->142A	0.10158
131B ->137B	0.40778
131B ->146B	0.11784
132B ->138B	0.16740
132B ->141B	-0.15904
132B ->142B	0.12835
133B ->139B	-0.16762
133B ->141B	-0.12863
133B ->142B	-0.15896
134B ->140B	0.29019
135B ->138B	-0.21286
135B ->142B	0.14688
136B ->139B	-0.21280
136B ->141B	0.14678

TTM-1TPE-2Cz (Optimized ground state structure, UB3LYP/6-31G (d, p))

Excited State 1: 2.060-A 1.6233 eV 763.80 nm f=0.0013 <S**2>=0.811

238B ->239B 0.99684

Excited State 2: 2.074-A 1.8516 eV 669.62 nm f=0.0935 <S**2>=0.825

237B ->239B 0.98396

Excited State 3: 3.465-A 2.3678 eV 523.62 nm f=0.0000 <S**2>=2.751

236A ->246A 0.11844

239A ->240A -0.59161

239A ->241A -0.13092

239A ->242A -0.21606

239A ->246A -0.12358

236B ->246B -0.11807

238B ->240B 0.63456

238B ->241B -0.11635

238B ->246B 0.12329

Excited State 4: 2.084-A 2.4768 eV 500.59 nm f=0.0001 <S**2>=0.836

236B ->239B 0.99025

Excited State 5: 2.216-A 2.6632 eV 465.55 nm f=0.0156 <S**2>=0.977

237A ->241A -0.17002

238A ->241A -0.22589

238A ->242A 0.12433

226B ->239B -0.21215

228B ->239B -0.27505

229B ->239B -0.11039

230B ->239B 0.25637

231B ->239B 0.78068

Excited State 6: 2.233-A 2.7578 eV 449.58 nm f=0.0000 <S**2>=0.996

237A ->242A -0.10740

238A ->240A 0.14633

238A ->241A -0.14246

238A ->242A -0.25309

225B ->239B 0.35730

228B ->239B -0.12702

229B ->239B 0.75730

230B ->239B 0.10880

233B ->239B 0.16063

237B ->239B -0.12537

Excited State 7: 2.120-A 2.8803 eV 430.45 nm f=0.0032 <S**2>=0.873

238A ->241A 0.14875

226B ->239B 0.37994

227B ->239B 0.47674

228B ->239B 0.58142

231B ->239B 0.42049

Excited State 8: 2.141-A 2.9434 eV 421.22 nm f=0.0025 <S**2>=0.896

238A ->240A -0.10714

238A ->241A 0.16123

238A ->242A 0.14363

225B ->239B -0.48732

226B ->239B 0.54526

227B ->239B -0.22141

228B ->239B -0.30729

229B ->239B 0.39023

235B ->239B -0.14152

Excited State 9: 2.066-A 2.9495 eV 420.35 nm f=0.0001 <S**2>=0.817

233B ->239B -0.22260

234B ->239B 0.46805

235B ->239B 0.83109

Excited State 10: 2.135-A 2.9580 eV 419.15 nm f=0.0033 <S**2>=0.889

238A ->242A	-0.20020
225B ->239B	0.53257
226B ->239B	0.54445
227B ->239B	-0.40699
228B ->239B	-0.11408
229B ->239B	-0.33488
231B ->239B	0.10893

TTM-2TPE-2Cz (Optimized ground state structure, UB3LYP/6-31G (d, p))

Excited State 1: 2.058-A 1.6694 eV 742.69 nm f=0.0008 <S**2>=0.809

339B -> 341B 0.26982

340B -> 341B 0.95903

Excited State 2: 2.059-A 1.6704 eV 742.23 nm f=0.0005 <S**2>=0.809

339B -> 341B 0.95941

340B -> 341B -0.27104

Excited State 3: 2.077-A 1.8604 eV 666.44 nm f=0.1201 <S**2>=0.828

338B -> 341B 0.98172

Excited State 4: 2.069-A 1.9295 eV 642.57 nm f=0.0326 <S**2>=0.821

337B -> 341B 0.98495

Excited State 5: 3.471-A 2.3947 eV 517.74 nm f=0.0000 <S**2>=2.762

335A -> 349A 0.12068

339A -> 342A 0.24994

339A -> 343A 0.21621

339A -> 345A 0.13268

340A -> 342A 0.30741

340A -> 343A 0.26959

340A -> 345A 0.16726

341A -> 342A -0.19642

341A -> 343A -0.17136

341A -> 345A -0.10958

335B -> 349B -0.11104

339B -> 342B -0.51767

339B -> 343B -0.35300

339B -> 345B -0.13262

339B -> 349B 0.11740

Excited State 6: 3.471-A 2.3986 eV 516.91 nm f=0.0000 <S**2>=2.761

336A -> 350A -0.12136

339A -> 342A	0.15235
339A -> 343A	-0.19992
340A -> 342A	-0.24600
340A -> 343A	0.32024
340A -> 344A	0.15758
340A -> 345A	-0.13782
341A -> 342A	-0.19021
341A -> 343A	0.25187
341A -> 344A	0.12530
341A -> 345A	-0.10978
336B -> 350B	0.11616
340B -> 342B	0.33615
340B -> 343B	-0.52474
340B -> 344B	-0.16160
340B -> 350B	-0.12071
